# Five new species of *Macrothele* Ausserer, 1871 from China (Araneae, Macrothelidae)

**DOI:** 10.3897/zookeys.1052.68623

**Published:** 2021-07-30

**Authors:** Yejie Lin, Xunyou Yan, Shuqiang Li, Francesco Ballarin, Haifeng Chen

**Affiliations:** 1 Hebei Key Laboratory of Animal Diversity, College of Life Science, Langfang Normal University, Langfang 065000, China Langfang Normal University Langfang China; 2 Institute of Zoology, Chinese Academy of Sciences, Beijing 100101, China Institute of Zoology, Chinese Academy of Sciences Beijing China; 3 Systematic Zoology Laboratory, Department of Biological Sciences, Tokyo Metropolitan University, 1-1 Minami-Osawa, 192-0397, Tokyo, Japan Tokyo Metropolitan University Tokyo Japan

**Keywords:** Asia, cryptic species, mygalomorphae, taxonomy

## Abstract

Five new species of the genus *Macrothele* Ausserer, 1871 are described from China: *Macrothele emei* Lin & Li, **sp. nov.** (♂♀, Sichuan), *M. hanfeii* Lin & Li, **sp. nov.** (♂♀, Hainan), *M. hungae* Lin & Li, **sp. nov.** (♂♀, Taiwan), *M. limenghuai* Lin & Li, **sp. nov.** (♂♀, Sichuan), and *M. nanning* Lin & Li, **sp. nov.** (♂♀, Guangxi). Types of the new species are deposited in the Institute of Zoology, Chinese Academy of Sciences in Beijing, China.

## Introduction

**Figure 1.**
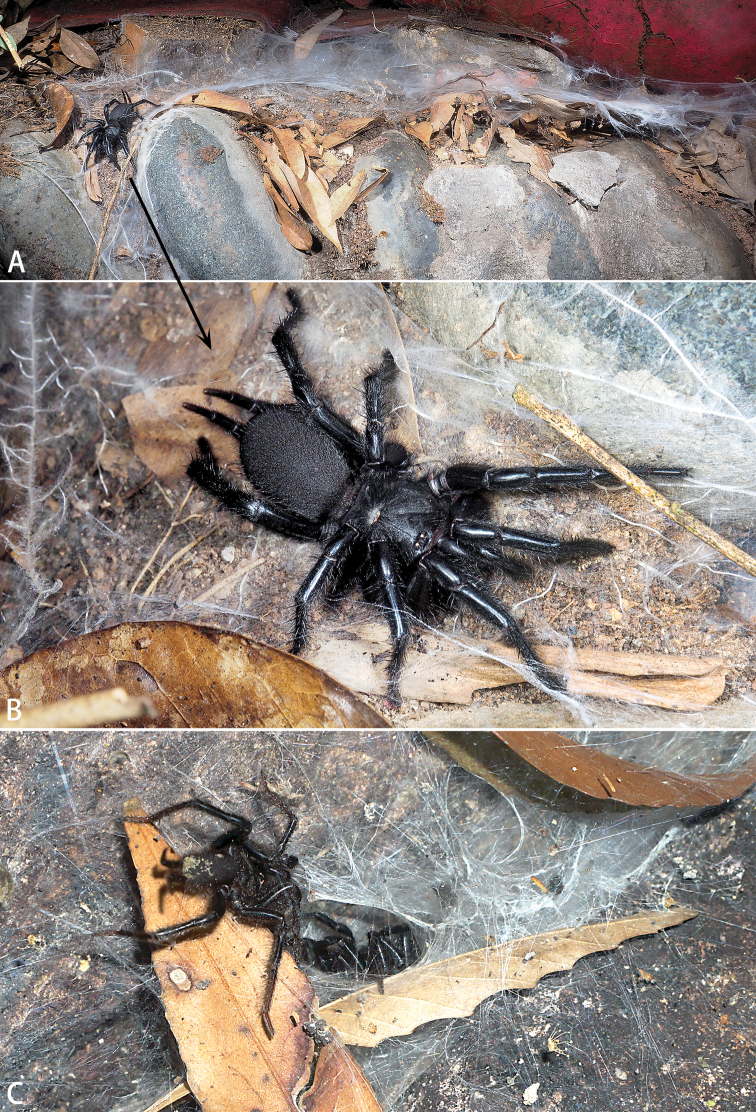
Live *Macrothele* spp. in situ **A, B***M. limenghuai* sp. nov., female **C***M. hanfeii* sp. nov., male and female, courtship. Photo by Xu Zhang (**A, B**) and Jiaxiang Wu (**C**).

The spider family Macrothelidae Simon, 1892 includes 40 species in one genus, *Macrothele* Ausserer, 1871 (Li 2020; WSC 2021). The majority of *Macrothele* species are distributed in Asia (33 species), but seven species are known from Europe and Africa. *Macrothele* commonly make webs in crevices or cavities in roadside soil slopes and occasionally build webs within and on leaf litter (Fig. 1). Here we describe five new *Macrothele* species from China.

## Materials and methods

All specimens were preserved in 75% ethanol at Institute of Zoology, Chinese Academy of Sciences (**IZCAS**) in Beijing, China. Spermathecae were cleared in trypsin enzyme solution to dissolve non-chitinous tissues. Specimens were examined under a LEICA M205C stereomicroscope. Photomicroscope images were taken with an Olympus C7070 zoom digital camera (7.1 megapixels). Laboratory habitus photographs were taken with a Canon EOS 60D digital camera equipped with a Canon EF 100 mm f/2.8L IS USM macro lens. Photos were stacked with Helicon Focus v. 6.7.1 and processed in Adobe Photoshop CC 2018. The terminology used in the text and figures follows Zhu and Song (2000). Distribution maps were generated using ArcMap v. 10.2.

All measurements are in millimeters. Total size does not include chelicerae. Eye sizes were measured as the maximum diameter from either the dorsal or frontal view. Leg measurements are given as follows: total length (femur, patella+tibia, metatarsus, tarsus). Abbreviations: AER, anterior eye row; ALE, anterior lateral eyes; AME, anterior median eyes; CD, copulatory ducts; PER, posterior eye row; PLE, posterior lateral eyes; PLS, posterior lateral spinnerets; PME, posterior median eyes; PMS, posterior median spinnerets; T, terminus of receptacula.

## Taxonomy

### Family Macrothelidae Simon, 1892

#### Genus Macrothele

Taxon classificationAnimaliaAraneaeMacrothelidae

Ausserer, 1871

C7E19C52-5819-53CA-9B2B-97C400B2ADCF

##### Type species.

*Mygale calpeiana* Walckenaer, 1805

#### Macrothele emei

Taxon classificationAnimaliaAraneaeMacrothelidae

Lin & Li sp. nov.

92B263B3-001E-55C9-8FD6-FAF5961073F1

http://zoobank.org/9DDA3C10-942E-4ADB-8C17-E33E09CCF3BB

**Figure 2.**
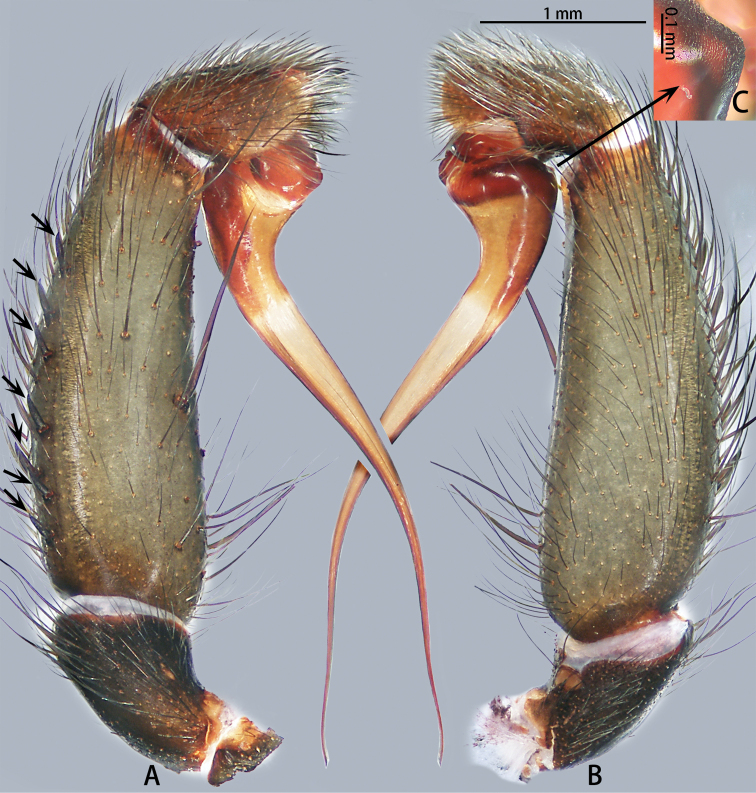
*Macrothele emei* sp. nov., left palp, holotype **A** prolateral view **B** retrolateral view **C** bulb apophysis. Black arrows pointing to the conical spines; black arrow to inset pointing to the close up of rough-textured apophysis.

**Figure 3.**
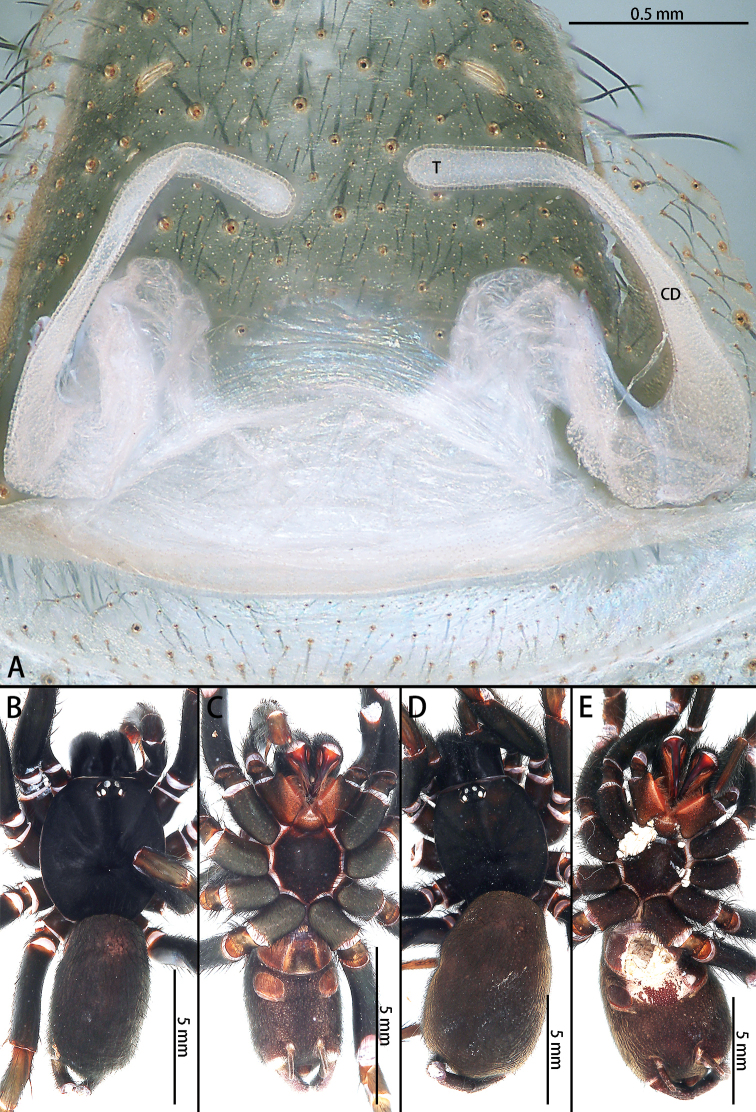
*Macrothele emei* sp. nov., female genitalia, male holotype and female paratype **A** female genitalia, ventral view **B** male habitus, dorsal view **C** male habitus, ventral view **D** female habitus, dorsal view **E** female habitus, ventral view. Abbreviations: **CD** copulatory ducts, **T** terminus of receptacula.

Figures 2 , 3 , 13A , 14A , 16

##### Type material.

***Holotype***: 1♂ (IZCAS-Ar41850) China, Sichuan Province, Mount Emei, Shengshuige to Huayan Temple, 29.5697°N, 103.4100°E, elevation ca 830 m, 29.IX.2016, Zhe Zhao & Xiaoqing Zhang leg. ***Paratypes***: 1♂3♀ (IZCAS-Ar41851–Ar41854), same data as holotype; 1♀ (IZCAS-Ar41855), China, Sichuan Province, Mount Emei, east of Dapingshanzhuang, Yuanhong Cave, 29.5688°N, 103.4089°E, elevation ca 850 m, 29.IX.2016, Zhe Zhao & Xiaoqing Zhang leg.; 1♂1♀ (IZCAS-Ar41856–Ar41857), China, Sichuan Province, Mount Emei, Zhongfeng Old Temple to Shengshuige, 29.5701°N, 103.4029°E, elevation ca 790 m, 28.IX.2016, Zhe Zhao & Xiaoqing Zhang leg.

##### Etymology.

The specific epithet refers to the type locality; noun in apposition.

##### Diagnosis.

Males of *Macrothele emei* sp. nov. resemble those of *M. digitata* Chen, Jiang & Yang, 2020 and *M. palpator* Pocock, 1901 in having the conical spines dorsally on palpal tibia and the embolus of the same shape. Females of the new species resemble *M. palpator* by the G-shaped receptacula with long copulatory ducts. Males of *M. emei* sp. nov. can be distinguished from *M. digitata* and *M. palpator* by the presence of seven short spines visible in dorsal view on the distal third of the palpal tibia in dorsal view (vs 13–15 long spines in *M. digitata* and in *M. palpator*). Females of *M. emei* sp. nov. can be differentiated from *M. palpator* by the ratio of the length of the copulatory ducts after the second turn almost 1:2 and the second turn expanded (vs length ratio of the copulatory duct folds 1:3, second fold unexpanded in *M. palpator*).

##### Description.

**Male (holotype)** (Figs 2, 3B, C): Total length 21.53, carapace 8.52 long, 6.93 wide; opisthosoma 12.51 long, 5.52 wide. Carapace dark brown, covered with short setae. Fovea deep, round. AER slightly procurved, PER recurved. Eye sizes and interdistances: AME 0.25, ALE 0.36, PME 0.28, PLE 0.27; AME–AME 0.13, ALE–AME 0.09, ALE–PLE 0.11, PLE–PME 0.08, PME–PME 0.27. Cheliceral promargin with 15 stout teeth, basomesally with 20 denticles. Labium brown, with ca 124 cuspules; sternum chestnut, with three pairs of sigillae. Legs dark brown. Leg measurements: I 19.75 (6.52 + 7.01 + 5.13 + 3.10), II 22.88 (6.02 + 7.11 + 5.24 + 4.51), III 23.55 (6.51 + 7.92 + 5.01 + 4.11), IV 30.21 (8.11 + 9.10 + 8.00 + 5.00). Leg formula: 4321. Abdomen dark brown, hairy. Spinnerets: PMS one segment, 2.22 long, 0.51 wide, PMS–PMS 1.60; PLS three segments. PLS 12.28 long (3.66, 3.38, 3.54). Tip of PLS white.

***Male palp*** (Figs 2, 13A, 14A). Maxillae with ca 153 cuspules. Palpal trochanter without lyral spines. Bulb nearly globose, with a ventral apophysis with a rough texture; embolus flat, slightly curved, needle-shaped, and expanded basally.

**Female** (Fig. 3A, D, E): total length 22.40, carapace 9.54 long, 8.82 wide; opisthosoma 11.31 long, 6.07 wide. Eye sizes and interdistances: AME 0.20, ALE 0.39, PME 0.29, PLE 0.36; AME–AME 0.08, ALE–AME 0.12, ALE–PLE 0.08, PME–PME 0.62, PLE–PME 0.07. Cheliceral promargin with 14 stout teeth, basomesally with 21 denticles. Endites brown, labium with ca 118 cuspules. Palpal trochanter without lyral spines. Leg measurements: I 19.76 (5.42 + 6.71 + 4.63 + 3.12), II 20.04 (6.20 + 7.10 + 3.74 + 3.00), III 21.26 (6.10 + 6.92 + 5.03 + 3.21), IV 25.37 (7.11 + 8.21 + 6.13 + 3.92). Leg formula: 4321. Abdomen dark brown, hairy. Spinnerets: PMS one segment, 2.53 long, 0.71 wide, PMS–PMS 2.25; PLS three segments. PLS 12.28 long (3.83, 3.72, 4.73).

***Female genitalia*** (Fig. 3A) simple, with two turns. Receptacula directed anteriorly, turning laterally, then dorsally, finally medially; somewhat G-shaped. The length ratio of the duct after the lateral turn to the dorsal turn is 2:1.

##### Variations.

Male total length 14.21–21.53 (*n* = 3), female total length 13.52–22.40 (*n* = 5).

##### Distribution.

China (Sichuan).

#### Macrothele hanfeii

Taxon classificationAnimaliaAraneaeMacrothelidae

Lin & Li sp. nov.

C80A331E-E97C-5ECA-8CAB-7133E82EE518

http://zoobank.org/874E5529-D0DB-413E-B4B7-78810FEAA607

**Figure 4.**
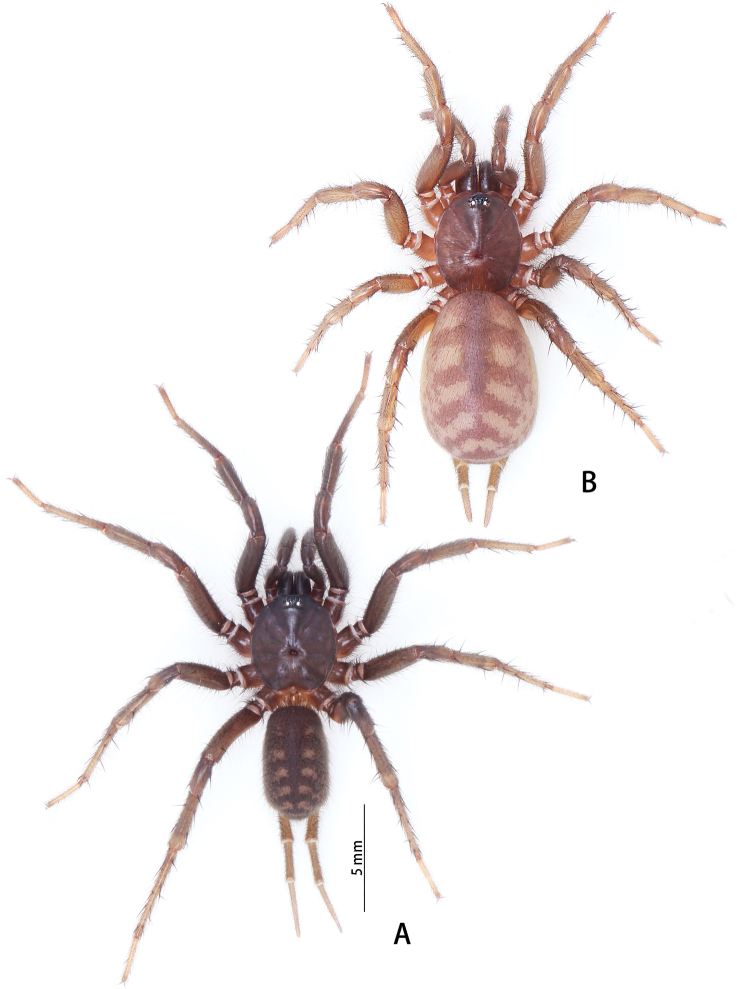
Live *Macrothele hanfeii* sp. nov., paratypes **A** male **B** female.

**Figure 5.**
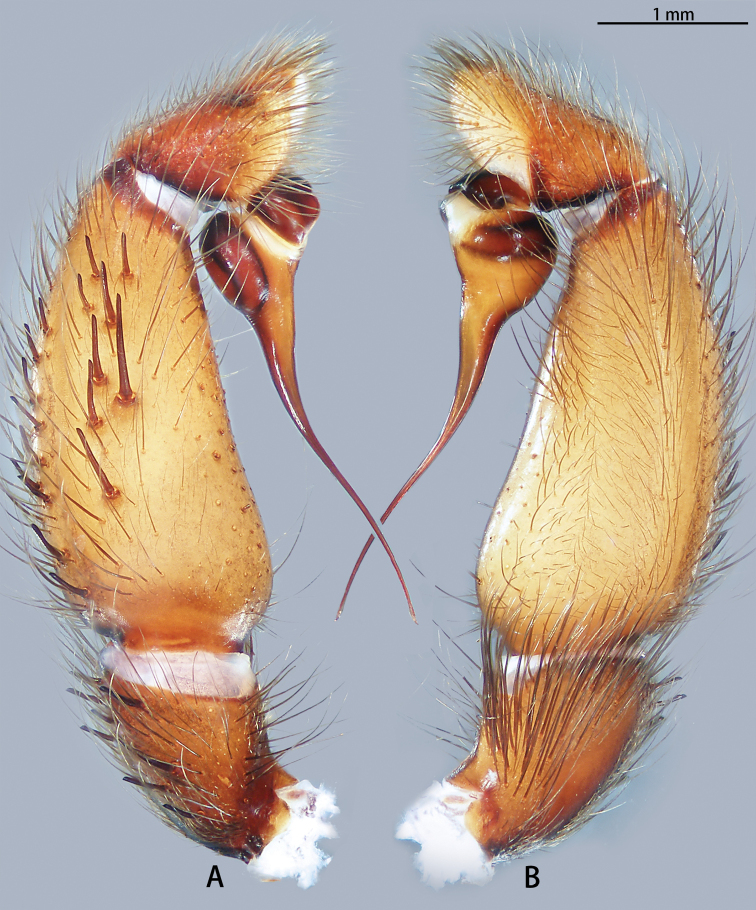
*Macrothele hanfeii* sp. nov., left palp, holotype **A** prolateral view **B** retrolateral view.

**Figure 6.**
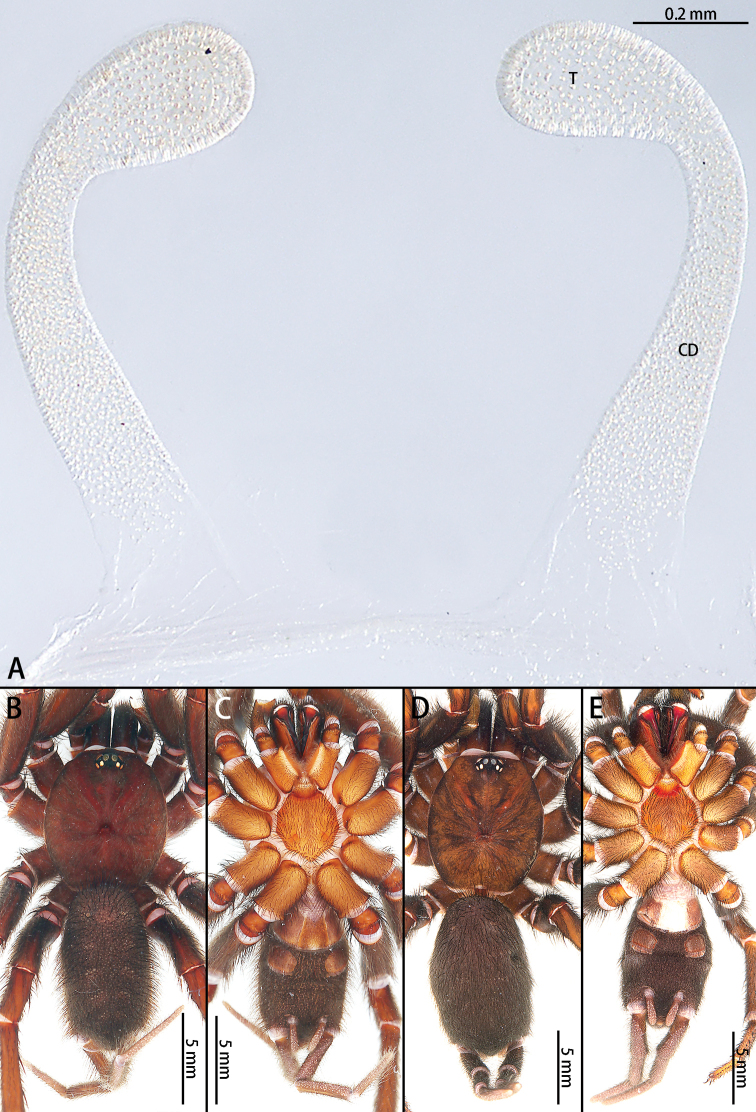
*Macrothele hanfeii* sp. nov., female genitalia, male holotype, and female paratype **A** female genitalia, ventral view **B** male habitus, dorsal view **C** male habitus, ventral view **D** female habitus, dorsal view **E** female habitus, ventral view. Abbreviations: **CD** copulatory ducts, **T** terminus of receptacula.

Figures 4 , 5 , 6 , 13F , 14F , 16

##### Type material.

***Holotype***: 1♂ (IZCAS-Ar41857) China, Hainan Province, Ledong Li Autonomous County, Jianfengling National Park, Mingfeng Valley, 18.7417°N, 108.8416°E, elevation ca 980 m, 21.IV.2018, Yejie Lin & Jiaxiang Wu leg. ***Paratypes***: 1♀ (IZCAS-Ar41858), same data as holotype; 3♀ (IZCAS-Ar41859–Ar41861), same data as holotype but 23.XI.2020, Yunhu Mo leg.

##### Etymology.

The species epithet refers to Mr Hanfei Gao who helped with this research; noun (name) in genitive case.

##### Diagnosis.

Male of *Macrothele hanfeii* sp. nov. resemble those of *M. holsti* Pocock, 1901 and *M. multispine* Wang, Li & Yang, 2019 by having similar bulb shape. Females of *M. hanfeii* sp. nov. resemble the aforementioned species by the apically globose receptacula that bend inwards. Male of *M. hanfeii* sp. nov. can be distinguished from *M. holsti* and *M. multispine* in that the palpal tibia bear seven blunt spines in prolateral view and nine in dorsal view that extend onto the patella and a large spine present in the center of the area with blunt spines (vs 14 blunt spines in prolateral view and 13 in dorsal view in *M. holsti*, and three blunt spines in prolateral view and one in dorsal view in *M. multispine*; large spine absent in *M. holsti* and *M. multispine*, and in *M. multispine*, the blunt spines are absent on the patella). Females differ from those of *M. holsti* and *M. multispine* by the short, robust copulatory ducts and the receptacula apically teardrop shaped (vs copulatory ducts long and thin and receptacula apically oval in *M. holsti* and *M. multispine*).

##### Description.

**Male (holotype)** (Figs 4A, 5, 6B, C, 13F, 14F): total length 20.03, carapace 8.02 long, 7.13 wide; opisthosoma 14.01 long, 5.12 wide. Carapace dark brown, glabrous, covered with short setae, middle of cephalic region with row of setae. Fovea deep, round. AER slightly procurved, PER recurved. Eye sizes and interdistances: AME 0.35, ALE 0.46, PME 0.31, PLE 0.32; AME–AME 0.15, ALE–AME 0.11, ALE–PLE 0.12, PLE–PME 0.05, PME–PME 0.31. Cheliceral promargin with 13 stout teeth, basomesally with 19 denticles. Labium brown, with ca 33 cuspules; sternum chestnut, with three pairs of sigillae. Legs dark brown. Leg measurements: I 21.75 (6.52 + 7.01 + 5.13 + 3.10), II 22.88 (6.02 + 7.11 + 5.24 + 4.51), III 23.55 (6.51 + 7.92 + 5.01 + 4.11), IV 30.21 (8.11 + 9.10 + 8.00 + 5.00). Leg formula: 4321. Abdomen dark brown, hairy. Spinnerets: PMS one segment, 1.76 long, 0.48 wide, PMS–PMS 1.36; PLS three segments. PLS 3.94 long (3.19, 3.56, 4.50).

***Male palp*** (Figs 5, 13F, 14F). Maxillae with ca 70 cuspules. Palpal trochanter without lyral spines. Patella with eight blunt spines, tibia with blunt spines, seven in prolateral view and nine in dorsal view. Bulb nearly globose; embolus slightly curved and needle-shaped.

**Female** (Figs 4B, 6A, D, E): total length 17.20, carapace 7.62 long, 8.52 wide; opisthosoma 12.21 long, 8.11 wide. Eye sizes and interdistances: AME 0.22, ALE 0.43, PME 0.38, PLE 0.40; AME–AME 0.15, ALE–AME 0.09, ALE–PLE 0.05, PME–PME 0.58, PLE–PME 0.05. Cheliceral promargin with 15 stout teeth, basomesally with 31 denticles. Palpal trochanter without lyral spines. Endites brown, labium with ca 32 cuspules. Leg measurements: I 17.76 (5.02 + 6.51 + 4.13 + 3.10), II 20.04 (6.20 + 7.10 + 3.74 + 3.00), III 21.26 (6.10 + 6.92 + 5.03 + 3.21), IV 25.37 (7.11 + 8.21 + 6.13 + 3.92). Leg formula: 4321. Abdomen dark brown, hairy. Spinnerets: PMS one segment, 2.18 long, 0.53 wide, PMS–PMS 2.38; PLS three segments. PLS 11.60 long (3.52, 3.76, 4.32).

***Female genitalia*** (Fig. 6A) simple. Receptacula apically teardrop shaped, copulatory ducts short and robust, the ratio of the length of the receptacula apically to the length of the copulatory ducts is almost 1:2.

##### Variation.

Female total length 17.20–22.52 (*n* = 4).

##### Distribution.

China (Hainan).

#### Macrothele hungae

Taxon classificationAnimaliaAraneaeMacrothelidae

Lin & Li sp. nov.

45280282-5B9B-5D3E-BFD9-B75D72ACC964

http://zoobank.org/C38C581D-3167-4B06-B5D0-58B4278B0CEC

**Figure 7.**
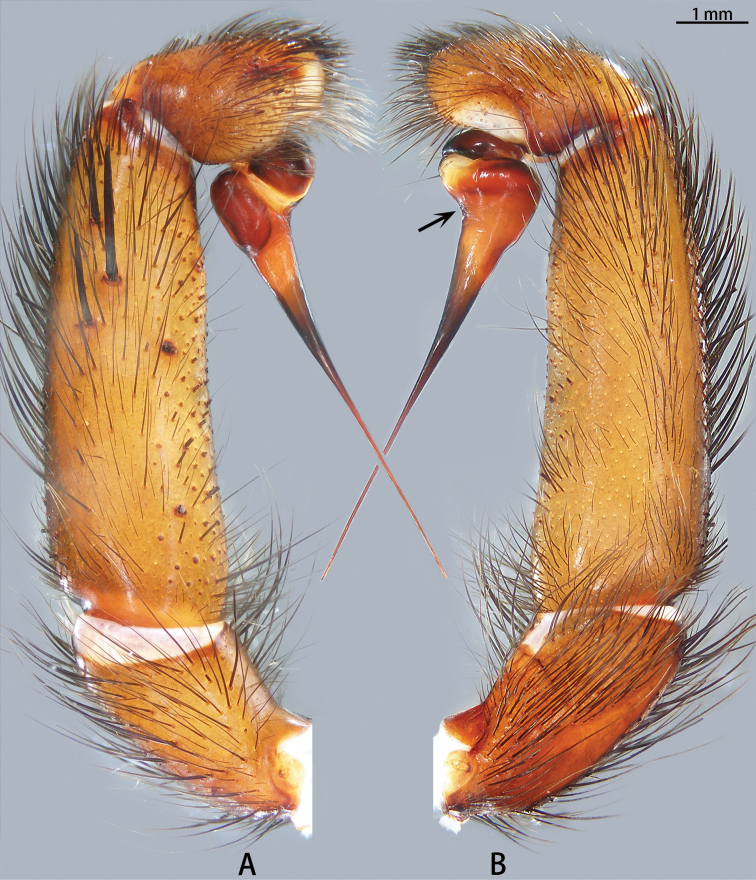
*Macrothele hungae* sp. nov., left palp, holotype **A** prolateral view **B** retrolateral view. Black arrow: expansion of embolic base. Black arrow pointing to the expansion of embolic base.

**Figure 8.**
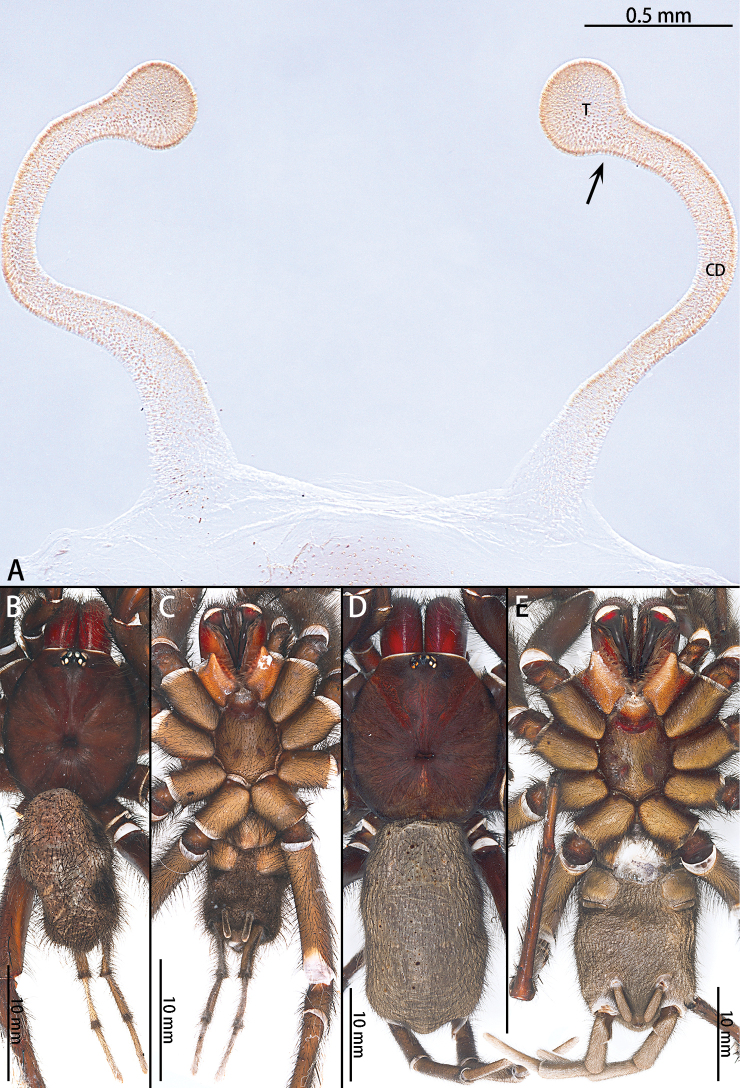
*Macrothele hungae* sp. nov., female genitalia, male holotype and female paratype **A** female genitalia, ventral view **B** male habitus, dorsal view **C** male habitus, ventral view **D** female habitus, dorsal view **E** female habitus, ventral view. Black arrow pointing to the width of the terminus. Abbreviations: **CD** copulatory ducts, **T** terminus of receptacula.

Figures 7 , 8 , 13E , 14E , 15B, F , 16

Macrothele gigas Haupt, 2008: 20, fig. 4B (♀, misidentified).

##### Type material.

***Holotype***: 1♂ (IZCAS-Ar41862) China, Taiwan, Kenting National Forest Recreation Area, Pingtung County, Kenting, 27.VI. 2013, Yanzhou Tong leg. Paratypes: 3♀ (IZCAS-Ar41863–Ar41865), same data as holotype.

##### Species studied for comparison.

*Macrothele gigas* Shimojana & Haupt, 1998: 3♂, Japan, Ishigaki Island, Yarabudake, 24.4405°N, 124.0874°E, 10.IV.2019, Sibagarasu Kurosaki leg.; 3♀, same data as male but 8.IX.2019.

##### Etymology.

The specific epithet is dedicated to Ms Hung Hsiu-chu, the first elected chairwoman of the Kuomintang in Taiwan, China; noun (name) in genitive case.

##### Diagnosis.

*Macrothele hungae* sp. nov. resembles *M. gigas* (Figs 13D, 14D, 15C, D) by the large size, the needle-shaped embolus of the male, and the apically globose receptacula and the S-shaped copulatory ducts of the female. Male of *M. hungae* sp. nov. can be distinguished from those of *M. gigas* by three rows of 22–25 lyral spines on the maxillae, palpal tibia with six spines in prolateral view, and embolic base expansion begins at one of the apical thirds of the bulb (vs 11 lyral spines in two rows, tibia with 1 or 2 spines in prolateral view, expansion of embolic base begins at the halfway point of the bulb). Females can be differentiated from those of *M. gigas* by having the base of copulatory ducts as wide as the receptacula and the receptacula apically oval (vs the ratio of the width of the base of copulatory ducts to the width of the terminus almost 2:3, and the receptacula apically teardrop shaped in *M. gigas*).

##### Description.

**Male (holotype)** (Figs 7, 8B, C, 13E, 14E, 15B, F): total length 25.63, carapace 13.22 long, 12.23 wide; opisthosoma 15.89 long, 9.89 wide. Carapace dark brown, covered with short setae, middle of cephalic region with a row of setae. Fovea deep, round. AER slightly procurved, PER recurved. Eye sizes and interdistances: AME 0.50, ALE 0.76, PME 0.41, PLE 0.48; AME–AME 0.25, ALE–AME 0.12, ALE–PLE 0.16, PLE–PME 0.07, PME–PME 0.88. Chelicerae red, promargin with 14 stout teeth, basomesally with 22 denticles. Labium brown, with ca 104 cuspules; sternum chestnut, with three pairs of sigillae. Legs dark brown. Leg measurements: I 43.07 (10.11 + 15.83 + 10.20 + 6.93), II 48.44 (12.22 + 16.61 + 11.10 + 8.51), III 41.44 (10.31 + 14.00 + 10.91 + 6.22), IV 61.05 (17.52 + 17.11 + 19.20 + 7.22). Leg formula: 4213. Abdomen dark brown, hairy. Spinnerets: PMS one segment, 3.12 long, 0.67 wide, PMS–PMS 1.55; PLS three segments. PLS 14.10 long (4.30, 4.70, 5.10).

***Male palp*** (Figs 7, 13E, 14E). Maxillae with ca 115 cuspules. Palpal trochanter with 25 lyral spines. Tibia with six spines. Bulb nearly globose; embolus needle shaped, expanded at the base.

**Female** (Fig. 8A, D, E): total length 40.81, carapace 18.22 long, 16.52 wide; opisthosoma 22.61 long, 13.13 wide. AER slightly procurved, PER recurved. Eye sizes and interdistances: AME 0.52, ALE 0.93, PME 0.72, PLE 0.58; AME–AME 0.31, ALE–AME 0.20, ALE–PLE 0.12, PME–PME 1.16, PLE–PME 0.13. Cheliceral promargin with 11 stout teeth, basomesally with 28 denticles. Endites brown, labium with ca 118 cuspules. Leg measurements: I 59.54 (16.21 + 22.74 + 12.30 + 8.29), II 61.97 (16.40 + 22.21 + 14.93 + 8.43), III 54.81 (13.50 + 20.12 + 14.32 + 6.87), IV 66.02 (17.14 + 22.13 + 18.83 + 7.92). Leg formula: 4213. Abdomen dark brown, hairy. Spinnerets: PMS one segment, 5.12 long, 0.91 wide, PMS–PMS 4.42; PLS three segments. PLS 22.29 long (7.35, 7.51, 7.43).

***Female genitalia*** (Fig. 8A) simple. Receptacula apically oval; copulatory duct narrow, S-shaped; width of the base of the copulatory ducts and the width of the terminus are nearly equal.

##### Variation.

Female total length 40.81–51.40 (*n* = 3).

##### Distribution.

China (Taiwan).

#### Macrothele limenghuai

Taxon classificationAnimaliaAraneaeMacrothelidae

Lin & Li sp. nov.

7F773E37-867B-50B5-81D0-D54EABFCCDB0

http://zoobank.org/5A2112F7-B00F-49BB-B662-A16CE5A56B2D

**Figure 9.**
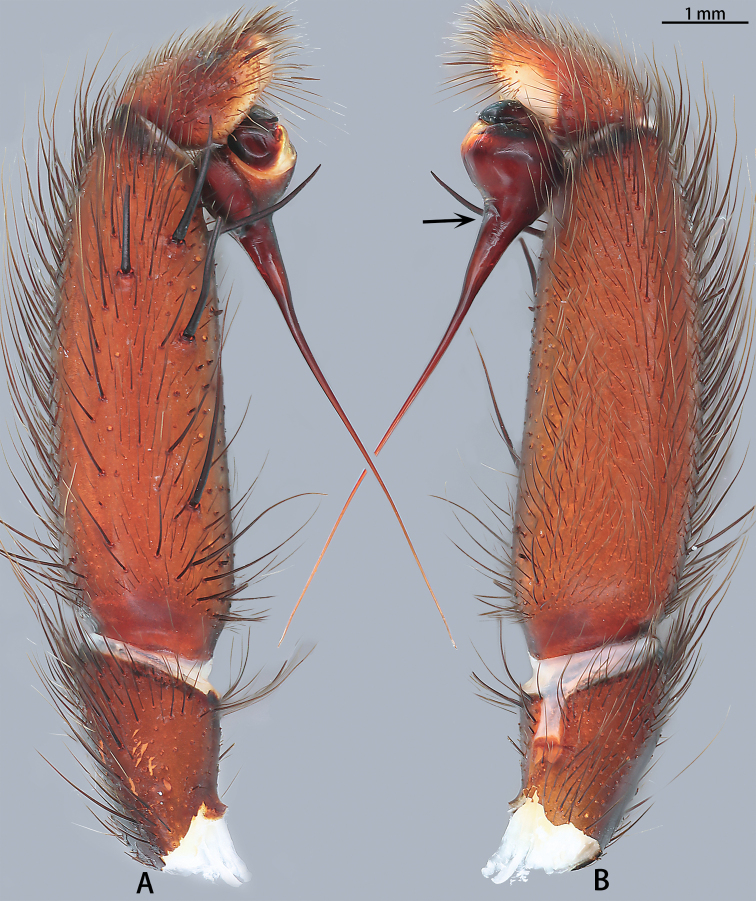
*Macrothele limenghuai* sp. nov., left palp, holotype **A** prolateral view **B** retrolateral view. Black arrow pointing to the depression on the base of the embolus.

**Figure 10.**
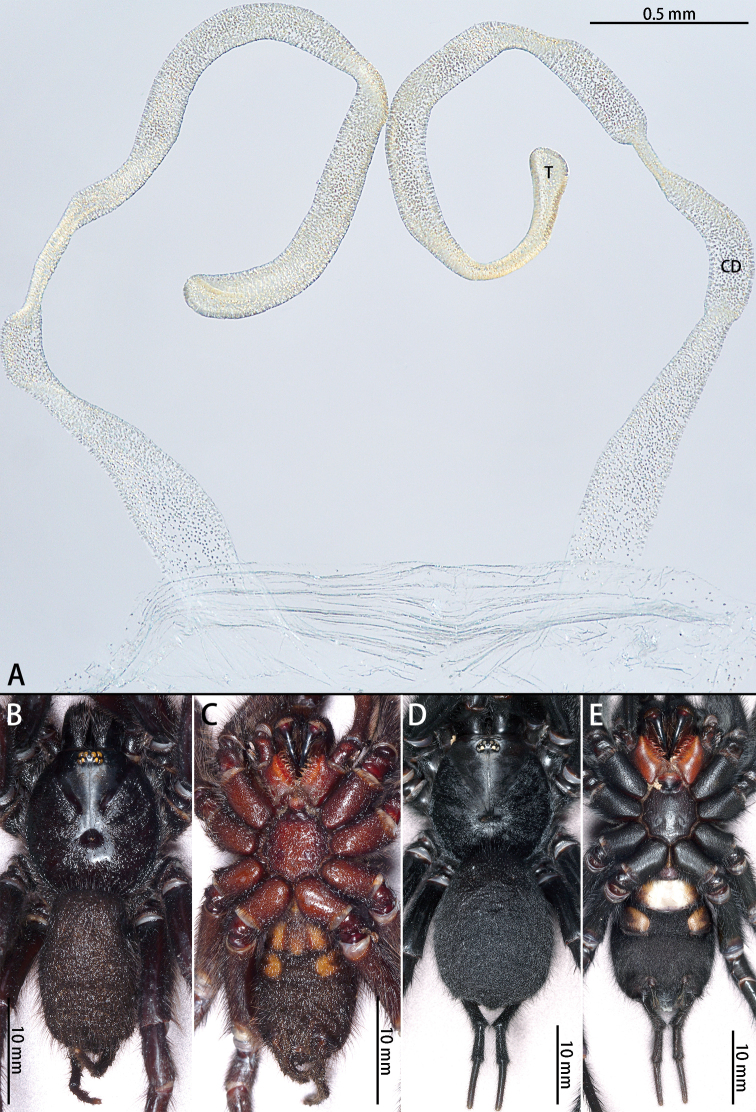
*Macrothele limenghuai* sp. nov., female genitalia, male holotype, and female paratypes **A** female genitalia, ventral view **B** male habitus, dorsal view **C** male habitus, ventral view **D** female habitus, dorsal view **E** female habitus, ventral view. Abbreviations: **CD** copulatory ducts, **T** terminus of receptacula.

Figures 9 , 10 , 13B , 14B , 15A, E , 16

##### Type material.

***Holotype***: 1♂ (IZCAS-Ar41866) China, Sichuan Province, Ya’an, Yucheng District, Sichuan Agriculture University, Laoban Mountain region, 29.9757°N, 102.9932°E, 17.VI.2014, Yejie Lin leg. ***Paratypes***: 1♂ (IZCAS-Ar41867), same data as holotype; 1♀ (IZCAS-Ar41868), same data as holotype but 22.X.2020, Menghua Li leg.

##### Etymology.

The species epithet is for Mr Menghua Li who collected the paratype; noun (name) in genitive case.

##### Diagnosis.

Males of *Macrothele limenghuai* sp. nov. resemble those of *M. monocirculata* Xu & Yin, 2000 and *M. raveni* Zhu, Li & Song, 2000 by having similar palpal bulb morphology, but they can be distinguished by having five tibial spines visible in prolateral view, the embolus as long as the tibia, and the base of the embolus with a depression (vs tibia with four spines in *M. monocirculata* or three spines in *M. raveni* visible in prolateral view, embolus notably longer than tibia and without basal depression). Females of *Macrothele limenghuai* sp. nov. resemble those of *M. monocirculata* and *M. raveni* by the receptacula coiling almost 360°, but they can be differentiated by the copulatory ducts bending outward medially (120°) (vs copulatory ducts straight in *M. monocirculata* and bent 90° in *M. raveni*).

##### Description.

**Male (holotype)** (Figs 9, 10B, C, 13B, 14B, 15A, E): total length 28.13, carapace 13.86 long, 12.43 wide; opisthosoma 15.19 long, 9.52 wide. Carapace dark brown, covered with short setae, middle of cephalic region with row of setae. Fovea deep, round. AER slightly procurved, PER recurved. Eye sizes and interdistances: AME 0.43, ALE 0.65, PME 0.42, PLE 0.51; AME–AME 0.31, ALE–AME 0.13, ALE–PLE 0.16, PLE–PME 0.11, PME–PME 0.89. Chelicerae black, promargin with 11 stout teeth, basomesally with 27 denticles. Labium brown, with ca 104 cuspules; sternum chestnut, with three pairs of sigillae. Legs dark brown. Leg measurements: I 44.26 (12.31 + 13.63 + 11.10 + 7.22), II 46.30 (11.81 + 16.23 + 11.11 + 7.15), III 41.66 (11.10 + 12.74 + 11.60 + 6.22), IV 53.85 (13.62 + 17.83 + 14.29 + 8.11). Leg formula: 4213. Abdomen dark brown, hairy. Spinnerets: PMS one segment, 1.44 long, 0.56 wide, PMS–PMS 2.35; PLS three segments. PLS 12.18 long (4.08, 4.16, 4.10).

***Male palp*** (Figs 9, 13B, 14B). Maxillae with ca 172 cuspules. Palpal trochanter with 20 lyral spines. Tibia with five spines. Bulb nearly globose; embolus needle shaped; embolus base with depression.

**Female** (Fig. 10A, D, E): total length 39.30, carapace 15.14 long, 27.31 wide; opisthosoma 24.11 long, 14.26 wide. Eye sizes and interdistances: AME 0.44, ALE 0.77, PME 0.47, PLE 0.59; AME–AME 0.36, ALE–AME 0.14, ALE–PLE 0.15, PLE–PME 0.08. Cheliceral promargin with 11 stout teeth, basomesally with 28 denticles. Endites brown, labium with ca 127 cuspules. Palpal trochanter with lyral spines. Leg measurements: I 46.07 (13.01 + 17.03 + 11.02 + 5.02), II 47.24 (12.08 + 18.10 + 11.01 + 6.05), III 45.32 (11.07 + 15.08 + 12.05 + 6.12), IV 54.32 (14.10 + 19.02 + 14.09 + 7.11). Leg formula: 4213 Abdomen dark brown, hairy. Spinnerets: PMS one segment, 1.44 long, 0.53 wide, PMS–PMS 1.61; PLS three segments. PLS 13.63 long (2.40, 4.63, 4.48).

***Female genitalia*** (Fig. 10A) simple. Receptacula apically expanded; copulatory ducts narrow, C-shaped, coiled anteriorly from 180° to 300°.

##### Variation.

Male total length 28.13–32.62 (*n* = 2).

##### Distribution.

China (Sichuan).

#### Macrothele nanning

Taxon classificationAnimaliaAraneaeMacrothelidae

Lin & Li sp. nov.

C12C0551-E1B9-5D9F-A3F5-4A9611135E33

http://zoobank.org/722973E8-94A2-4656-82AC-E3528E277854

**Figure 11.**
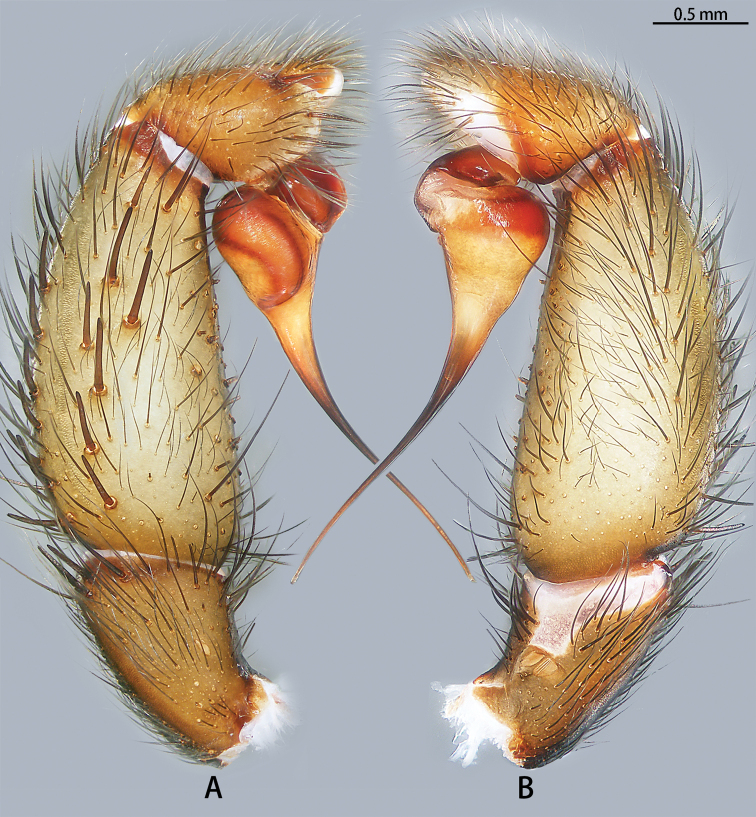
*Macrothele nanning* sp. nov., left palp, holotype **A** prolateral view **B** retrolateral view.

**Figure 12.**
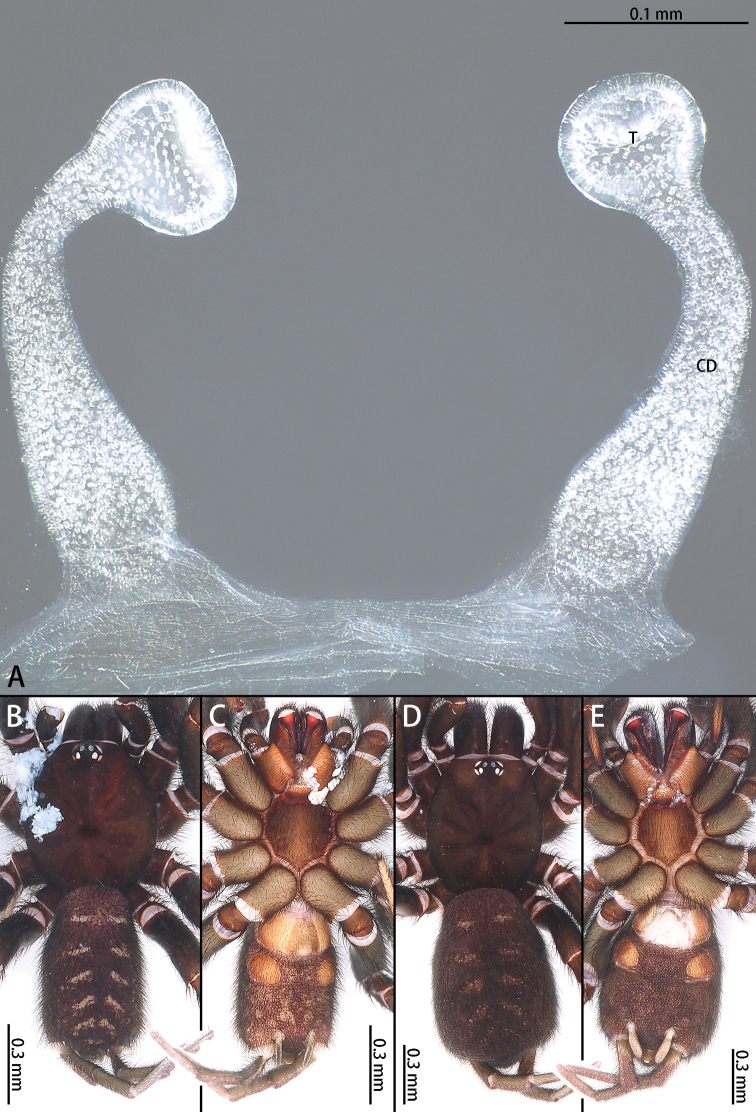
*Macrothele nanning* sp. nov., female genitalia, male holotype, and female paratypes **A** female genitalia, ventral view **B** male habitus, dorsal view **C** male habitus, ventral view **D** female habitus, dorsal view E female habitus, ventral view. Abbreviations: CD copulatory ducts, T terminus of receptacula.

**Figure 13.**
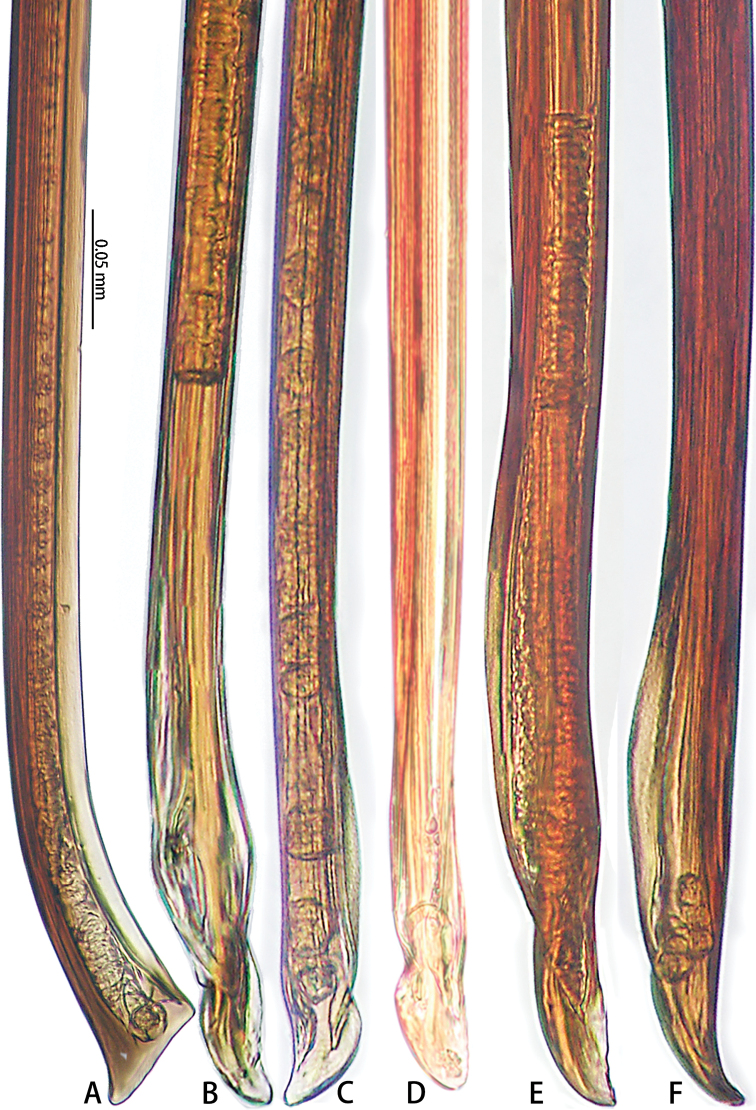
Prolateral view of embolic tips of six species of *Macrothele***A***M. emei* sp. nov. **B***M. limenghuai* sp. nov. **C***M. nanning* sp. nov. **D***M. gigas***E***M. hungae* sp. nov. **F***M. hanfeii* sp. nov.

**Figure 14.**
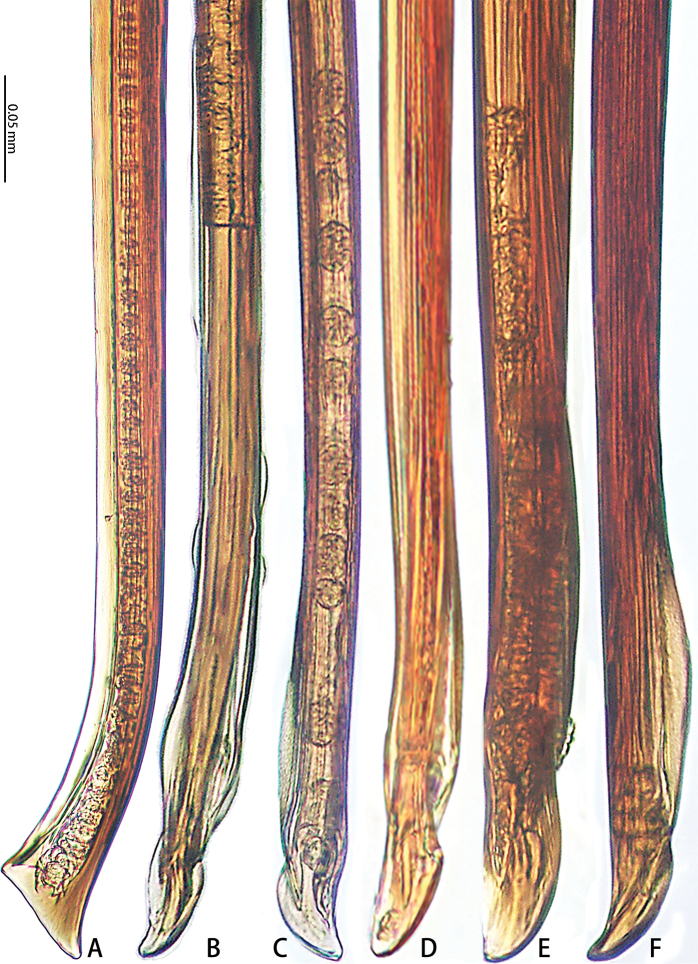
Retrolateral view of embolic tips of six species of *Macrothele***A***M. emei* sp. nov. **B***M. limenghuai* sp. nov. **C***M. nanning* sp. nov. **D***M. gigas***E***M. hungae* sp. nov. **F***M. hanfeii* sp. nov.

Figures 11 , 12 , 13C , 14C , 16

##### Type material.

***Holotype***: 1♂ (IZCAS-Ar41869) China, Guangxi Zhuang Autonomous Region, Nanning, Suxu Town, Mu Village, Shibaluohan Cave, 22.5433°N, 108.0565°E, elevation ca 190 m, 09.V.2015, Zhigang Chen & Yunchun Li leg. ***Paratypes***: 5♀ (IZCAS-Ar41870–Ar41874), same data as holotype.

##### Etymology.

The species epithet refers to the type locality; noun in apposition.

##### Diagnosis.

Males of *Macrothele nanning* sp. nov. resemble *M. multispine* by having blunt spines in lateral and dorsal views of palpal tibia and similar palpal bulb morphology, and females of the new species are similar to others by the apically globose receptacula bent inwards apically. Male of *M. nanning* sp. nov. can be distinguished from *M. multispine* by having the tibia with eight blunt spines in prolateral view and seven in dorsal view, the blunt spines extending onto patella (vs three blunt spines in prolateral view and one in dorsal view and blunt spines absent from patella). Females can be differentiated from *M. multispine* by the short, robust copulatory ducts and receptacula expanded basally (vs copulatory ducts long and narrow and receptacula base unexpanded).

##### Description.

**Male (holotype)** (Figs 11, 12B, C, 13C, 14C): total length 12.63, carapace 5.30 long, 5.26 wide; opisthosoma 7.31 long, 4.17 wide. Carapace dark brown, covered with short setae, middle of cephalic region with row of setae. Fovea deep, round. AER slightly procurved, PER recurved. Eye sizes and interdistances: AME 0.25, ALE 0.47, PME 0.26, PLE 0.28; AME–AME 0.14, ALE–AME 0.06, ALE–PLE 0.56, PLE–PME 0.04, PME–PME 0.44. Cheliceral promargin with 11 stout teeth, basomesally with 17 denticles. Labium brown, with ca 17 cuspules; sternum chestnut, with three pairs of sigillae. Legs dark brown. Leg measurements: I 16.61 (4.49 + 5.77 + 3.91 + 2.44), II 17.30 (4.99 + 5.64 + 4.23 + 2.44), III 16.48 (4.17 + 5.13 + 4.74 + 2.44), IV 20.64 (5.06 + 6.73 + 6.09 + 2.76). Leg formula: 4231. Abdomen dark brown, hairy. Spinnerets: PMS one segment, 1.02 long, 0.24 wide, PMS–PMS 0.99; PLS three segments. PLS 3.94 long (2.45, 2.20, 2.78).

***Male palp*** (Figs 11, 13C, 14C). Maxillae with ca 43 cuspules. Palpal trochanter without lyral spines. Patella with three blunt spines; tibia with blunt spines, eight in prolateral view and seven in dorsal view. Bulb nearly globose; embolus slightly curved, needle shaped, and expanded at base.

**Female** (Fig. 12A, D, E): total length 17.08, carapace 7.12 long, 6.09 wide; opisthosoma 9.81 long, 6.86 wide. Eye sizes and interdistances: AME 0.23, ALE 0.50, PME 0.22, PLE 0.41; AME–AME 0.20, ALE–AME 0.12, ALE–PLE 0.08, PME–PME 0.64, PLE–PME 0.04. Cheliceral promargin with 13 stout teeth, basomesally with 22 denticles. Endites brown, labium with ca 30 cuspules. Leg measurements: I 18.28 (6.09 + 6.41 + 3.65 + 2.13), II 15.57 (5.00 + 5.45 + 2.88 + 2.24), III 21.26 (4.81 +5.00 + 4.10 + 2.37), IV 20.51 (5.45 + 7.18 + 5.32 + 2.56). Leg formula: 4132. Abdomen dark brown, hairy. Spinnerets: PMS one segment, 1.76 long, 0.49 wide, PMS–PMS 1.72; PLS three segments. PLS 7.81 long (2.36, 2.38, 3.06).

***Female genitalia*** (Fig. 12A) simple. Receptacula apically oval; copulatory ducts short and robust, expanded; the ratio of the length of the receptacula apically to the length of the copulatory duct is almost 1:4.

##### Variation.

Female total length 12.43–17.08 (*n* = 5).

##### Distribution.

China (Guangxi).

**Figure 15.**
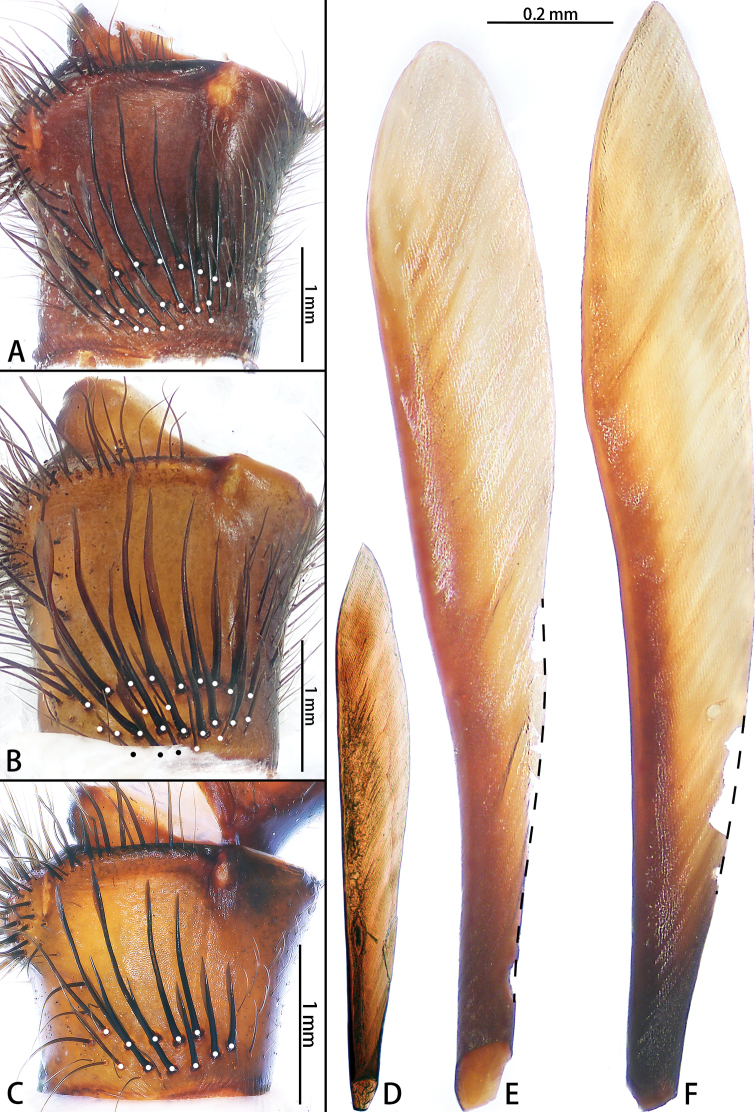
Male left palpal trochanter, retrolateral view, and lyral setae **A, E***Macrothele limenghuai* sp. nov. **B, F***M. hungae* sp. nov. **C, D***M. gigas*.

**Figure 16.**
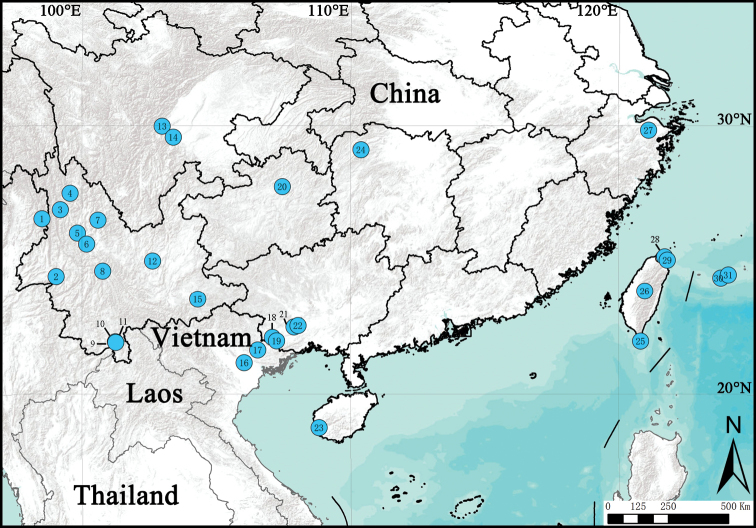
Distribution records of *Macrothele* species in East Asia **1***M. yani***2***M. arcuata***3***M. jingzhao***4***M. yunlingensis***5***M. undata***6***M. cangshanensis***7***M. yongshengensis***8***M. jinlin***9***M. bannaensis***10***M. yunnanica***11***M. menglunensis***12***M. multispine***13***M. limenghuai* sp. nov. **14***M. emei* sp. nov. **15***M. sanheensis***16***M. decemnotata***17***M. proserpina***18***M. monocirculata***19***M. raveni***20***M. guizhouensis***21***M. digitata***22***M. nanning* sp. nov. **23***M. hanfeii* sp. nov. **24***M. hunanica***25***M. hungae* sp. nov. **26***M. holsti***27***M. palpator***28***M. taiwanensis***29***M. simplicata***30***M. yaginumai***31***M. gigas*.

## Supplementary Material

XML Treatment for Macrothele

XML Treatment for Macrothele emei

XML Treatment for Macrothele hanfeii

XML Treatment for Macrothele hungae

XML Treatment for Macrothele limenghuai

XML Treatment for Macrothele nanning
